# Early pregnancy with low β-hCG levels progressing to severe preeclampisa: a case report highlighting individualized management strategies

**DOI:** 10.3389/fmed.2026.1785157

**Published:** 2026-03-25

**Authors:** Lufeng Pang, Qiongfen Fang, Xiaoqian Pan, Heyun Ruan

**Affiliations:** 1 Department of Obstetrics and Gynecology, Minzu Hospital of Guangxi Zhuang Autonomous Region, Affiliated Minzu Hospital of Guangxi Medical University, Nanning, Guangxi, China; 2 Chongzuo Key Laboratory of Precision Research for Regional High-Incidence Tumors, Minzu Hospital of Guangxi Zhuang Autonomous Region, Affiliated Minzu Hospital of Guangxi Medical University, Nanning, Guangxi, China

**Keywords:** individualized management, low β-HCG levels, placental-deriveddiseases, precise pregnancy preservation, severe preeclampsia

## Abstract

**Introduction:**

β-Human chorionic gonadotropin (β-hCG) is a pregnancy hormone secreted by the syncytiotrophoblast layer of the placenta. It is related to fetal growth as well as the placenta, uterus, and fetal function. Low β-hCG in early pregnancy is closely associated with pathological pregnancies such as miscarriage, and its mechanism may be related to abnormal development of placental villi. Here we present a 35-year-old pregnant woman who, after natural conception, had low levels of β-hCG in early pregnancy. After treatment with recombinant human granulocyte colony-stimulating factor, low molecular heparin, and dydrogesterone to maintain the pregnancy, she started taking aspirin 100 mg daily at 12 weeks of pregnancy. At 20 weeks of pregnancy, due to significant edema, the aspirin dosage was adjusted to 150 mg. After personalized treatment adjustments, she developed severe preeclampsia at 32^+4^ weeks of pregnancy. After precise management, a good prognosis for both mother and baby was achieved.

**Conclusion:**

Early pregnancy with low β-hCG due to poor placental function may develop into severe preeclampsia in the third trimester. Clinicians should pay attention to this type of low β-hCG pregnant women, manage individualized medication, and promote maternal and infant safety.

## Introduction

Human chorionic gonadotropin (hCG) is a glycoprotein hormone composed of two distinct subunits: α and β (1). While the α -subunit is structurally similar to that of luteinizing hormone (LH), follicle-stimulating hormone (FSH), and thyroid-stimulating hormone (TSH), making it susceptible to cross-reactivity and interference from other circulating markers, the β -subunit possesses a unique carboxyl-terminal sequence (2). Functionally, the two subunits play different roles. The α -subunit is primarily responsible for receptor binding stability, whereas the β -subunit confers biological specificity and determines the hormone’s unique interaction with the LH/CG receptor. Consequently, β -hCG is the specific target for clinical detection to ensure accuracy and avoid false positives associated with total hCG assays (3).

The characteristics of β-hCG levels have been widely used clinically as a predictive indicator of successful pregnancy (4); it was recognized as early as 1940 that an early decline in hCG is associated with spontaneous abortion, while abnormal levels of hCG, especially low β-hCG levels, often indicate poor embryonic development and early pregnancy loss (5); it may also be related to adverse pregnancy outcomes such as pre-eclampsia, preterm birth, and fetal growth restriction (6). The state of abnormal β-hCG levels may be closely related to abnormal development of placental villi (7). In normal pregnancy, β-hCG helps promote angiogenesis, while this process is inhibited in pre-eclampsia. Therefore, low levels or abnormal fluctuations of β-hCG may indicate placental dysfunction (8), thereby increasing the risk of early pregnancy loss or late pregnancy pre-eclampsia. In this case report, we aim to report a rare case of early pregnancy with low β-hCG levels that later developed into early-onset severe pre-eclampsia, with regular monitoring and individualized management, adjusting medication according to the condition, early detection of complications, timely termination of pregnancy, and ultimately achieving a good outcome for both mother and child.

## Case presentation

**Figure 1:**
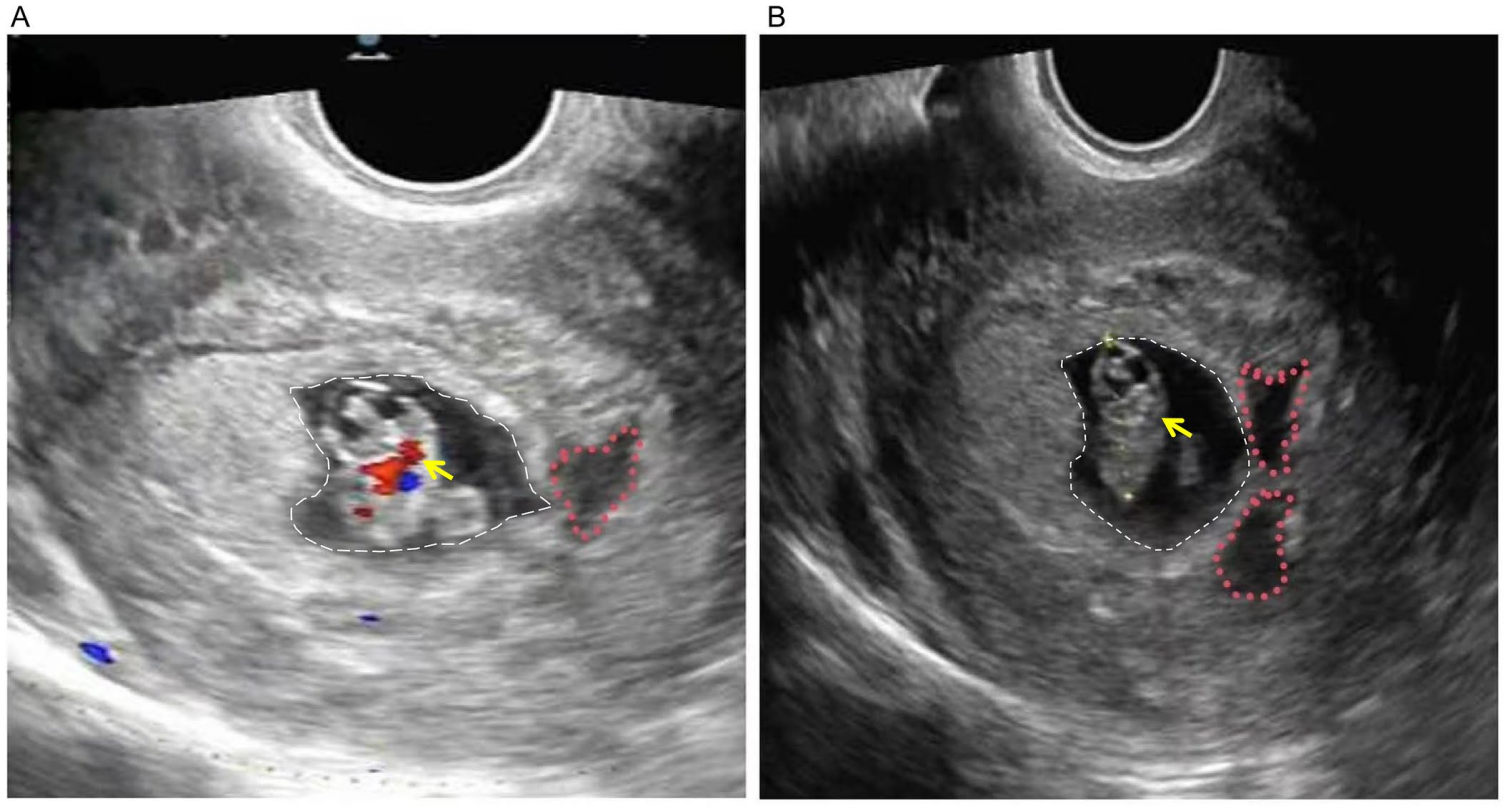
Transvaginal ultrasound in early pregnancy. **(A)** Color Doppler flow imaging (CDFI) reveals cardiac activity within the embryo (yellow arrow), indicated by the red and blue color signals, confirming fetal viability. The gestational sac is outlined by the white dashed line, while the adjacent subchorionic hematoma is marked by the red dotted line. **(B)** Corresponding two-dimensional grayscale ultrasound image showing the morphological relationship between the structures. The embryo (yellow arrow) is visualized within the gestational sac (white dashed line). A crescent-shaped anechoic area representing the subchorionic hematoma is clearly visible adjacent to the sac (red dotted line). Image acquired on February 12, 2025.

**Figure 2:**
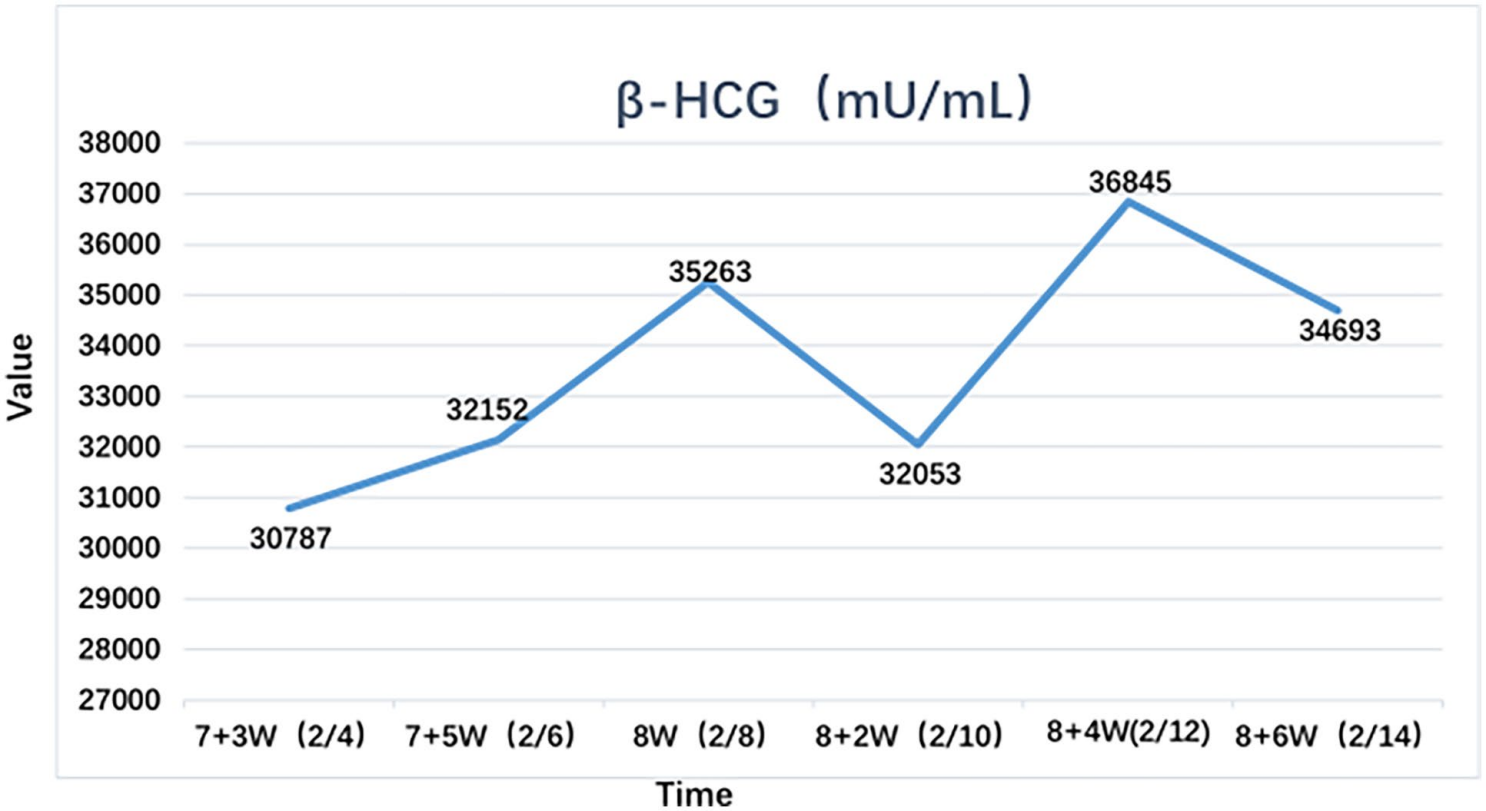
Serum β-hCG profile in early pregnancy. Sequential measurements of β**-**hCG (mIU/mL) from gestational week 7 + ^3^ to week 8 + ^6^. Sampling dates (month/day) are noted below each data point.

A 35-year-old woman with a pre-pregnancy body mass index (BMI) of 28.23, suffering from primary infertility for 2 years, conceived naturally after 2 years of pregnancy preparation. The last menstrual period (LMP) was on December 14, 2024. Ultrasound examinations performed at an external hospital at 7^+3^ weeks and 7^+5^ weeks of gestation confirmed that the crown-rump length (CRL) corresponded with the gestational age on both occasions. However, the measurements of gestational sac size, along with serum beta-human chorionic gonadotropin (β-hCG) levels, and progesterone, as assessed at an external hospital, all indicated values lower than the predicted values for the corresponding gestational age. Considering the risk of spontaneous abortion, oral administration of dydrogesterone was prescribed for tocolytic therapy to preserve the pregnancy; however, the therapeutic effect was unsatisfactory. She sought medical attention at 8 weeks of pregnancy. Given the patient’s two-year history of infertility and the current pregnancy complicated by unsatisfactory examination findings, coupled with her strong desire for a successful outcome, comprehensive evaluations were performed. Routine hematological and urinalysis tests were conducted. Furthermore, an extensive workup for potential etiologies of recurrent miscarriage and infertility was undertaken. This included screening for antinuclear antibodies (ANA) and related profiles, a full antiphospholipid syndrome (APS) panel (including lupus anticoagulant), and assays for Protein S, Protein C, and Antithrombin III. Thyroid function tests and infectious disease screenings were also performed. All investigations yielded normal results. Early pregnancy ultrasound revealed no uterine malformations. Neither partner’s chromosomal karyotype was tested. Since February 4th, to understand the development of the gestational sac and the effectiveness of treatment, β-hCG doubling has been monitored every other day (Table 1), and transvaginal B-ultrasound has been monitored as needed (Table 2). On February 12, 2025 (8 + 4 weeks of pregnancy), the difference between the average gestational sac measurement and the length of the fetal pole was measured to below 5 millimeters (Figure 1). Serial ultrasound evaluations (Table 2) revealed a persistent gestational sac-embryo discrepancy. Although the crown-rump length (CRL) increased normally from 17 mm to 21 mm, the mean sac diameter (MSD) remained relatively small. Notably, at 8^+4^ weeks, the MSD − CRL discrepancy narrowed to 4.7 mm. a low MSD-CRL difference signifies the heightened probability of miscarriage,the lower the MSD-CRL value, the higher the probability of miscarriage (9). Concurrently, the subchorionic hematoma showed significant reduction in size. Considering the high likelihood of embryo developmental issues and miscarriage, she was given subcutaneous injections of enoxaparin 4,000 AxaIU qd, and recombinant human granulocyte colony-stimulating factor (rhG-CSF) 0.15 mg was safely used every other day while monitoring routine blood tests, stopping the use of rhG-CSF after 9 weeks of pregnancy. During early pregnancy, β-hCG, progesterone, and estrogen levels rose slowly; after related treatment, the highest β-hCG value reached 36,745 mIU/mL at 8^+4^ weeks (Figure 2). By 12 weeks on March 5, 2025, placenta growth factor tests showed: PLGF 61.06 pg./mL, sFLT-12049.07 pg./nL, sFLT-1/PlGF 33.56; NT testing showed no abnormalities. Heparin and dydrogesterone were discontinued. Low-dose aspirin (LDA) 100 mg was administered orally once daily. At 20 weeks, the patient experienced bilateral lower limb edema (+) without dizziness, headache, or visual disturbances. Blood pressure was 110/68 mmHg. In the clinic, the patient had negative proteinuria, normal liver function, and treatment was switched to LDA 150 mg orally once daily. On July 30, 2025, during a check-up at 32 + 4 weeks of pregnancy, high blood pressure of 159/106 mmHg, urine protein 3+, and bilateral lower limb edema (++) were noted, with no abdominal pain, bloating, dizziness, headache, or blurred vision; considering preeclampsia, she was admitted. Ultrasound showed an intrauterine pregnancy with a single viable fetus in the head-down position, with fetal size corresponding to 31 weeks+. After admission, magnesium sulfate was administered for anticonvulsant treatment, and oral sustained-release nifedipine 20 mg was given every 8 h for antihypertensive treatment. Dexamethasone was used to promote fetal lung maturity. Due to age 35 years, pre-pregnancy BMI: 28.23, weight gain during pregnancy 26 kg, diagnosed with preeclampsia. VTE score 3 points, high risk of thrombosis. To prevent thrombosis, enoxaparin anticoagulant therapy is administered for thrombosis prevention (10, 11). After 2 days of treatment, the blood pressure remained high, with the highest blood pressure at admission being 185/115 mmHg. Upon admission at 32 + 4 weeks (July 30), enoxaparin was re-initiated due to a high VTE risk score (3 points) and severe preeclampsia, despite having been discontinued at 12 weeks. It was then discontinued again on July 31, 2025, 24 h prior to the planned cesarean section to minimize bleeding risk. It was planned to terminate the pregnancy on August 1, 2025, after completing one cycle of fetal lung maturation promotion. A cesarean section was performed at 17:35 on the same day, and a live male infant was delivered. The Apgar score was 1′-10 points, 5′-10 points, with no visible deformities, weighing 1,580 grams and measuring 42 cm in length. The placental volume was slightly small, measuring approximately 12 × 9 × 1.5 cm, with no other abnormalities observed. The infant was immediately transferred to the Neonatal Intensive Care Unit (NICU). Postoperatively, antihypertensive, anticonvulsant, sedation, and prevention of infection treatments were administered. There was minimal vaginal bleeding 24 h after cesarean delivery, and enoxaparin 4000AxaIU was continued to prevent thrombosis until discharge. Postoperative antihypertensive treatment included nifedipine 20 mg every 8 h and labetalol 100 mg every 12 h, with adjustments made based on blood pressure readings. Blood pressure stabilized after surgery and returned to normal within 12 days postpartum. After 15 days of stopping antihypertensive medication, blood pressure remained stable within the normal range. The patient was discharged on the 7th postoperative day. The newborn recovered well and was discharged 2 weeks later. Postpartum assessment at 3 months follow-up showed no signs or symptoms of any active systemic diseases in the patient, and the child was healthy. The management throughout the pregnancy is detailed in Table 3.

**Table 1:** Serial serum β-hCG levels during the observation period.

Date	Gestational age (weeks+days)	β-hCG level (mIU/mL)*	Reference range (mIU/mL)†	Status vs. Reference
Feb 4, 2025	7 + 3	30,787	3,697 – 163,563	Within range
Feb 6, 2025	7 + 5	32,152	3,697 – 163,563	Within range
Feb 8, 2025	8 + 0	35,263	32,065 – 149,571	Within range
Feb 10, 2025	8 + 2	32,053	32,065 – 149,571	Below lower limit
Feb 12, 2025	8 + 4	36,845	32,065 – 149,571	Within range
Feb 14, 2025	8 + 6	34,693	32,065 – 149,571	Within range

**Table 2:** Serial ultrasound measurements of gestational parameters and subchorionic hematoma resolution.

Date	Gestational age (weeks+days)	Gestational Sac dimensions (L × W × H, mm)	Mean Sac diameter (MSD, mm)*	Crown-Rump length (CRL, mm)	MSD − CRL discrepancy (mm)†	Subchorionic hematoma (dimensions or depth, mm)
Feb 10, 2025	8 + 2	25 × 24 × 23	24.0	17	7.0	28 × 14
Feb 12, 2025	8 + 4	24 × 32 × 18	24.7	20	4.7	15 × 7
Feb 15, 2025	9 + 0	28 × 32 × 23	27.7	21	6.7	Depth: 13

L, length; W, width; H, height; MSD, mean sac diameter; CRL, crown-rump length.

*MSD was calculated as (L + W + H)/3. †The MSD − CRL discrepancy indicates the difference between the sac size and embryonic size. A value < 10 mm often suggests a small gestational sac relative to the embryo.

**Table 3:** Clinical timeline, major events, and corresponding treatments during pregnancy.

Gestational week	Event	Treatment
7^+3^	Small gestational sac; delayed embryonic growth	Dydrogesterone 10 mg tid, oral
8 to 8^+6^	Ultrasound indicates: Early viable intrauterine gestation. Gestational sac-embryo discrepancy (5–7 mm). Intrauterine fluid.	Dydrogesterone 10 mg tid, oral Enoxaparin 4,000 AXaIU QD, subcutaneous injection rhG-CSF 0.15 mg QOD, subcutaneous injection
9 to 11^+6^	-	Dydrogesterone 10 mg tid, oral Enoxaparin 4,000 AXaIU QD, subcutaneous injection
12	Normal nuchal translucency (NT)	Aspirin 100 mg, oral
20	Lower extremity edema (+)	Aspirin 150 mg, oral
32^+4^	Preeclampsia	Aspirin discontinued; Hospitalization for antihypertensive therapy; Dexamethasone for fetal lung maturation
32^+6^	Preeclampsia, with severe hypertension (blood pressure up to 185/115 mmHg)	Cesarean section

tid, three times daily; QD, once daily; QOD, every other day.

## Discussion

Low serum β-hCG levels in early pregnancy are often associated with adverse outcomes, including miscarriage and ectopic pregnancy. Previous studies have demonstrated that persistently lowβ-hCG levels can indicate a non-viable pregnancy, complicating clinical decision-making. For instance, a study highlighted that low serumβ- hCG levels often correlate with a higher likelihood of early pregnancy loss, with significant implications for clinical management strategies (12–14). While persistently low or plateauing β-hCG trajectories typically signal a non-viable pregnancy, diagnostic challenges arise when fetal cardiac activity is present despite biochemical stagnation (15–17). Furthermore, emerging evidence suggests that abnormal β-hCG dynamics may not only reflect immediate viability but also serve as early markers for placental dysfunction and subsequent complications, such as severe preeclampsia (PE) (18). Our case presents a unique phenotype: a pregnancy that survived the first trimester despite a distinct β-hCG plateau and sonographic signs of restricted gestational sac growth, ultimately progressing to early-onset severe PE. This dissociation challenges the conventional view of low β-hCG as solely a predictor of miscarriage and highlights its potential role as an early warning sign for placental-mediated disorders.

In this case, serial evaluations revealed a significant discordance between embryonic somatic growth and trophoblastic function. As detailed in Table 1, while the crown-rump length (CRL) increased appropriately, the mean sac diameter (MSD) lagged, resulting in an MSD − CRL difference of only 4.7 mm at 8^+4^ weeks, The early miscarriage rate is significantly increased in pregnancies with an mGSD-CRL value less than 5 mm (9, 19). Concurrently, Table 2 demonstrates that maternal β-hCG levels exhibited a static plateau (fluctuating between 30,000 and 37,000 mIU/mL) rather than the expected exponential rise, with one value dipping marginally below the reference range. Notably, this biochemical and sonographic stagnation occurred despite confirmed fetal cardiac activity (Figure 1A). This specific pattern—viable embryonic growth coupled with compromised trophoblastic expansion and hormone production—suggests that the primary defect lay not in the embryo itself, but in the supporting placental unit.

This dissociation aligns with the “two-stage theory” of preeclampsia, where inadequate trophoblast invasion and impaired spiral artery remodeling in the first trimester (Stage 1) lead to placental hypoperfusion and oxidative stress, manifesting as clinical PE in later stages (Stage 2) (20–22). The observed low-normal β-hCG levels and small MSD are consistent with cohort studies linking first-trimester biomarkers to adverse placental outcomes (23). Mechanistically, β-hCG plays a regulatory role in trophoblast invasion; thus, its stagnation may reflect underlying molecular defects. Recent research by Wang et al. (24) identified specific tRNA-derived fragments (e.g., tiRNA-Gln-CTG) that regulate trophoblast function and correlate with hCG dynamics in PE. Although tiRNA levels were not measured in our patient, the observed β-hCG plateau strongly suggests a shared pathway of trophoblastic dysfunction. Therefore, the “successful” survival of the embryo in our case likely occurred despite a suboptimal placentation process, predisposing the patient to the severe PE observed later in gestation.

The management of such cases requires a paradigm shift from merely monitoring fetal viability to actively assessing placental health. In our patient, the administration of recombinant human granulocyte colony-stimulating factor (G-CSF) and low molecular weight heparin (LMWH) may have supported the compromised trophoblast, mitigating the immediate risk of miscarriage and allowing pregnancy continuation (25–27). However, the persistence of early warning signs (MSD-CRL discrepancy and β-hCG plateau) indicates that the risk of placental insufficiency remains elevated. Consequently, for patients presenting with this specific phenotype, conservative management in the first trimester should be followed by enhanced surveillance in the second and third trimesters. Protocols should include uterine artery Doppler velocimetry and serial growth scans to anticipate hypertensive disorders and fetal growth restriction. Early detection allows for timely interventions, potentially improving maternal and fetal outcomes in high-risk pregnancies characterized by low β-hCG trajectories.

However, the limitations of this case warrant careful consideration. While the findings highlight significant correlations, they are based on a single patient experience, which may not fully represent the broader population of women with low β-hCG levels. Future research should aim to conduct larger, multicentric studies to validate these observations and elucidate the mechanistic pathways linking β-hCG levels with pregnancy complications. Moreover, establishing comprehensive monitoring protocols and identifying biomarkers associated with adverse outcomes can enhance clinical management strategies, ultimately improving care for patients experiencing similar challenges.

## Data Availability

The original contributions presented in the study are included in the article/Supplementary material, further inquiries can be directed to the corresponding author/s.
