# Supplementary material for: Early pregnancy with low β-hCG levels progressing to severe preeclampisa: a case report highlighting individualized management strategies
Source: Front Med (Lausanne). 2026 Mar 25;13:1785157. doi: 10.3389/fmed.2026.1785157 (PMC13057494; doi:10.3389/fmed.2026.1785157)
Supplement: Supplementary file 1 [file Table_1.docx]

Table 1 Serial serum β-hCG levels during the observation period

| **Date** | **Gestational Age  (Weeks+Days)** | **β-hCG Level  (mIU/mL)*** | **Reference Range  (mIU/mL)†** | **Status vs. Reference** |
| --- | --- | --- | --- | --- |
| Feb 4, 2025 | 7 + 3 | 30,787 | 3,697 – 163,563 | Within range |
| Feb 6, 2025 | 7 + 5 | 32,152 | 3,697 – 163,563 | Within range |
| Feb 8, 2025 | 8 + 0 | 35,263 | 32,065 – 149,571 | Within range |
| Feb 10, 2025 | 8 + 2 | 32,053 | 32,065 – 149,571 | Below lower limit |
| Feb 12, 2025 | 8 + 4 | 36,845 | 32,065 – 149,571 | Within range |
| Feb 14, 2025 | 8 + 6 | 34,693 | 32,065 – 149,571 | Within range |
