# Supplementary material for: Early pregnancy with low β-hCG levels progressing to severe preeclampisa: a case report highlighting individualized management strategies
Source: Front Med (Lausanne). 2026 Mar 25;13:1785157. doi: 10.3389/fmed.2026.1785157 (PMC13057494; doi:10.3389/fmed.2026.1785157)
Supplement: Supplementary file 2 [file Table_2.docx]

Table 2  Serial ultrasound measurements of gestational parameters and subchorionic hematoma resolution

| **Date** | **Gestational Age  (Weeks+Days)** | **Gestational Sac Dimensions  (L × W × H, mm)** | **Mean Sac Diameter  (MSD, mm)*** | **Crown-Rump Length  (CRL, mm)** | **MSD − CRL  Discrepancy (mm)†** | **Subchorionic Hematoma  (Dimensions or Depth, mm)** |
| --- | --- | --- | --- | --- | --- | --- |
| Feb 10, 2025 | 8 + 2 | 25 × 24 × 23 | 24.0 | 17 | 7.0 | 28 × 14 |
| Feb 12, 2025 | 8 + 4 | 24 × 32 × 18 | 24.7 | 20 | 4.7 | 15 × 7 |
| Feb 15, 2025 | 9 + 0 | 28 × 32 × 23 | 27.7 | 21 | 6.7 | Depth: 13 |

Abbreviations: L, length; W, width; H, height; MSD, mean sac diameter; CRL, crown-rump length.
Notes:

*****MSD was calculated as (L+W+H)/3.
† The MSD − CRL discrepancy indicates the difference between the sac size and embryonic size. A value < 10 mm often suggests a small gestational sac relative to the embryo.
