# Supplementary material for: Early pregnancy with low β-hCG levels progressing to severe preeclampisa: a case report highlighting individualized management strategies
Source: Front Med (Lausanne). 2026 Mar 25;13:1785157. doi: 10.3389/fmed.2026.1785157 (PMC13057494; doi:10.3389/fmed.2026.1785157)
Supplement: Supplementary file 3 [file Table_3.docx]

Table 3 Clinical Timeline, Major Events, and Corresponding Treatments During Pregnancy

| Gestational Week | Event | Treatment |
| --- | --- | --- |
| 7^+3^ | Small gestational sac; delayed embryonic growth | Dydrogesterone 10 mg tid, oral |
| 8 to 8^+6^ | Ultrasound indicates:Early viable intrauterine gestation. Gestational sac-embryo discrepancy (5-7 mm). Intrauterine fluid. | Dydrogesterone 10 mg tid, oral Enoxaparin 4000 AXaIU QD, subcutaneous injection rhG-CSF 0.15 mg QOD, subcutaneous injection |
| 9 to 11^+6^ | - | Dydrogesterone 10 mg tid, oral Enoxaparin 4000 AXaIU QD, subcutaneous injection |
| 12 | Normal nuchal translucency (NT) | Aspirin 100 mg, oral |
| 20 | Lower extremity edema (+) | Aspirin 150 mg, oral |
| 32^+4^ | Preeclampsia | Aspirin discontinued; Hospitalization for antihypertensive therapy; Dexamethasone for fetal lung maturation |
| 32^+6^ | Preeclampsia, with severe hypertension (blood pressure up to 185/115 mmHg) | Cesarean section |

Note: tid = three times daily; QD = once daily;QOD=every other day.
